# Circulating innate lymphoid cells are dysregulated in patients with prostate cancer

**DOI:** 10.1186/s11658-025-00725-7

**Published:** 2025-04-18

**Authors:** Daniela Claudia Maresca, Evelina La Civita, Benedetta Romano, Maria Rosaria Ambrosio, Fabio Somma, Tania Wyss, Bernardo Rocco, Valentina Rubino, Luigi Cari, Philippe Krebs, Antonio Rodriguez-Calero, Matteo Ferro, Sara Trabanelli, Camilla Jandus, Felice Crocetto, Angela Ianaro, Daniela Terracciano, Giuseppe Ercolano

**Affiliations:** 1 Department of Pharmacy, School of Medicine, University of Naples Federico II, 80138 Naples, Italy; 2 Department of Translational Medical Sciences, University of Naples “Federico II”, Naples, Italy; 3 Institute for Experimental Endocrinology and Oncology “G. Salvatore”, National Research Council (IEOS-CNR), Via Pansini 5, 80131 Naples, Italy; 4 Translational Data Science-Facility, AGORA Cancer Research Center, Swiss Institute of Bioinformatics, Lausanne, Switzerland; 5 Department of Translational Medicine and Surgery, Gemelli IRCCS University Hospital Foundation in Rome, Università Cattolica del Sacro Cuore di Roma, Roma, Italy; 6 Department of Medicine and Surgery, University of Perugia, Perugia, Italy; 7 Institute of Tissue Medicine and Pathology, University of Bern, Bern, Switzerland; 8 Unit of Urology, Department of Health Science, University of Milan, ASST Santi Paolo and Carlo, Via A. Di Rudini 8, 20142 Milan, Italy; 9 Department of Pathology and Immunology, Faculty of Medicine, University of Geneva, Geneva, Switzerland; 10 Ludwig Institute for Cancer Research, Lausanne Branch, Lausanne, Switzerland; 11 Geneva Center for Inflammation Research, Geneva, Switzerland; 12 Translational Research Centre in Onco-Hematology (CRTOH), Geneva, Switzerland; 13 Department of Neurosciences, Reproductive Sciences and Odontostomatology, University of Naples “Federico II”, Naples, Italy; 14 Department of Pharmacy, School of Medicine, University of Naples Federico II, Via Domenico Montesano 49, 80131 Naples, Italy

**Keywords:** Prostate cancer, Innate lymphoid cells, ILC1s, ILC2s, IL-33, IL-18, IL-13

## Abstract

**Background:**

Prostate cancer (PCa) is the second most common cancer affecting men globally, especially those aged 50 years and above. Despite substantial progress in terms of both prognosis and therapy, PCa remains a significant health concern, necessitating the identification of novel therapeutic targets. Innate lymphoid cells (ILCs) have emerged as critical modulators of tumor immunity, exhibiting both pro- and antitumoral effects. However, little is known yet about their contribution in PCa. This study investigated the phenotypic and functional profiles of ILC subsets in the peripheral blood mononuclear cells (PBMCs) of patients with PCa stratified by Gleason score.

**Methods:**

PBMCs were isolated by Lymphoprep. ILC frequency and activity were evaluated by flow cytometry. The levels of ILC-activating cytokines were analyzed by multiplex assay in the serum of healthy donors (HDs) and patients with PCa. To evaluate the crosstalk between ILC2s and cancer cells, PC3 and DU145 human PCa cell lines were used.

**Results:**

We found a stage-dependent increase in the protumoral ILC2 frequency and a concurrent decrease in antitumoral ILC1s in patients with PCa compared with healthy controls. Interestingly, the frequency of ILC2s was higher in patients with elevated prostate-specific antigen (PSA) values, suggesting their potential as molecular predictor for defining the risk category of patients with PCa at diagnosis. Importantly, patients with PCa exhibited hyperactivated ILC2s, characterized by elevated interleukin (IL)-13 and IL-5 production, while ILC1s displayed reduced tumor necrosis factor (TNF)-α and interferon (IFN)-γ secretion. Furthermore, serum levels of ILC2-activating cytokines IL-33, IL-18, and prostaglandin D2 (PGD2) were elevated in patients with PCa. In vitro co-culture experiments demonstrated that PCa cell lines, capable of secreting these cytokines, could directly enhance ILC2 activity. Likewise, ILC2-derived IL-13 promoted PCa cell migration and invasion.

**Conclusions:**

Collectively, our findings highlight a dysregulated ILC profile in PCa, characterized by ILC2 dominance and heightened activity at the expense of ILC1s, suggesting both ILC1s and ILC2s as potential therapeutic targets for PCa treatment.

**Graphical Abstract:**

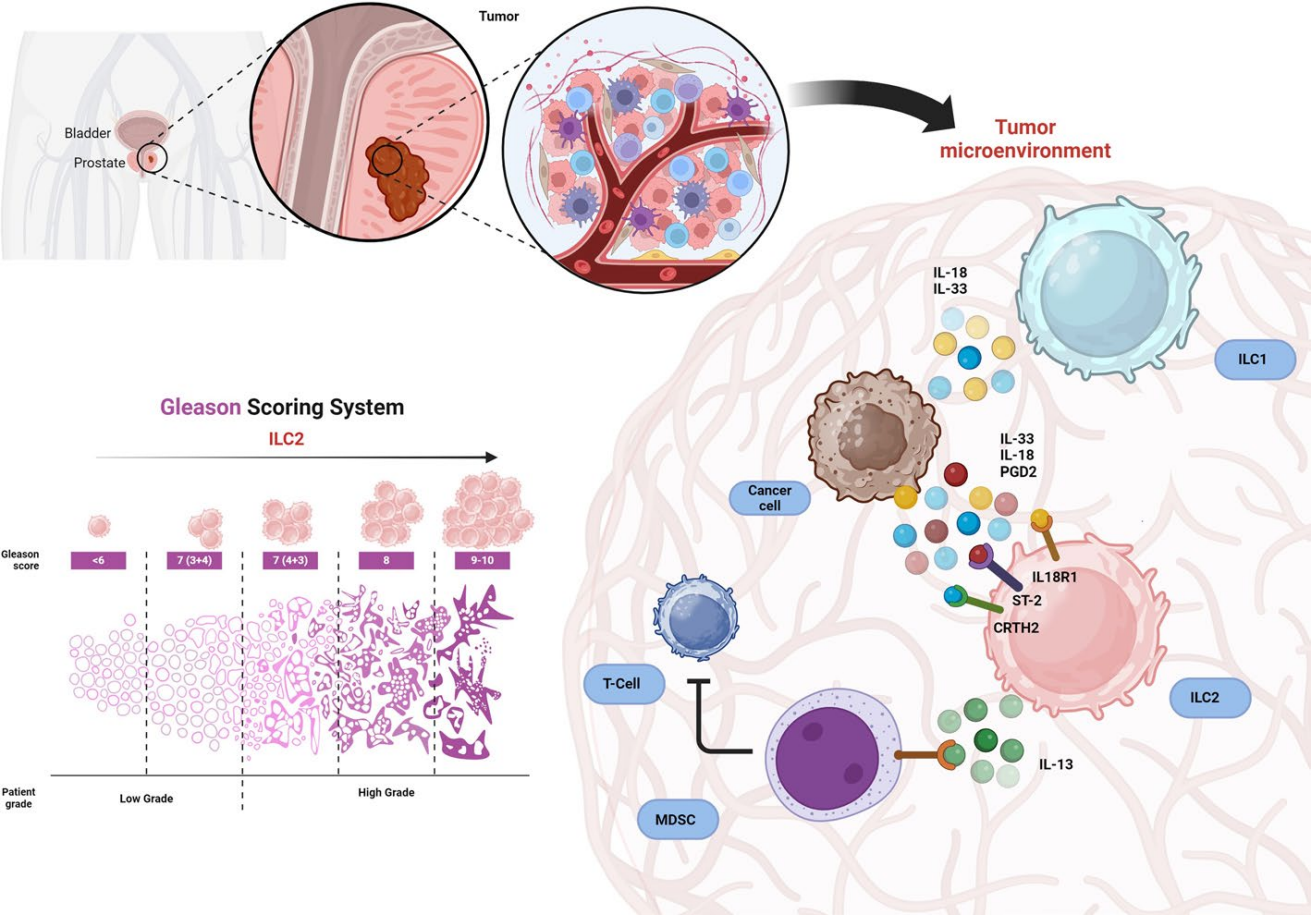

**Supplementary Information:**

The online version contains supplementary material available at 10.1186/s11658-025-00725-7.

## Introduction

According to the World Cancer Research Fund (WCRF), PCa is the second most common cancer affecting men worldwide. PCa mortality rate is different among regions, with a higher death rate in Western countries (USA, Europe, and Australia) [1]. Despite substantial progresses in terms of both prognosis and therapy, PCa still poses a major challenge to the healthcare system. In fact, early detection of PCa enhances the likelihood of successful treatment, subsequently leading to improved life expectancy for patients. In this context, PSA testing provides a diagnostic value for early detection of PCa [2]. Unfortunately, PSA levels are influenced by multiple events including benign prostatic hyperplasia (BPH), infections, sexual activity, exercise, and age [3–7]. Therefore, several new blood-based tests have been developed, such as the Prostate Health Index (PHI), 4K score, and Stockholm 3, which showed good ability to detect PCa [8]. However, these tests are not currently widely used in routine clinical practice, and PSA remains the most used tool available for the detection of PCa [9]. Nevertheless, the gold standard for the diagnosis and staging of PCa remains biopsy and the Gleason scoring system [10]. Although current therapies such as surgery, radiation, chemotherapy, hormone therapy, and immunotherapy have significantly reduced the mortality from this type of cancer over the years, a high percentage of patients still develop resistance [11]. Therefore, it may be clinically useful to identify novel therapeutic targets to fight chemotherapy resistance and reduce mortality rates. Among potential targets, ILCs are emerging as key players in orchestrating the pro- or antitumoral microenvironment in different types of cancer [12–14]. The different role of ILCs is strictly related to the subset considered (ILC1s, ILC2s, or ILC3s/Ps) as well as the tumor microenvironment and study models. In particular, ILC2s are mostly defined as a protumorigenic subset, given their type-2 immune functions through the secretion of IL-13, IL-5, IL-9, and the epidermal growth factor AREG, which has a detrimental role in various types of cancer [15–19]. Conversely, ILC1s play a crucial role in antitumor immunity by producing interferon-gamma (IFN-γ) and tumor necrosis factor-alpha (TNF-α) [20–23]. In human peripheral circulation, ILC precursors (ILCPs) have been recently discovered and have the potential to migrate to various tissues, where they can differentiate into mature ILCs based on local microenvironmental cues [24]. Importantly, ILC2-activating signals, including IL-33, IL-25, and thymic stromal lymphopoietin (TSLP), also contribute to the development of an immunosuppressive microenvironment in different types of cancer [25–28]. In the context of urologic cancers, such as prostate, kidney, bladder, and testicular cancers, few studies have focused on the characterization of ILC subsets. For instance, it has been demonstrated that ILC1s are increased but functionally exhausted in muscle‐invasive bladder cancer [29]. Conversely, ILC2s are increased and secrete IL-13 upon treatment with Bacillus Calmette-Guérin (BCG) vaccine [30]. Likewise, it has been reported that ILC2s are enriched in peripheral blood samples from patients with PCa [31]. Nevertheless, characterization of ILCs in PCa in terms of cytokine secretion and production, as well as the contribution of ILC-activating and ILC-secreted cytokines, is still limited. In this study, we investigated phenotypical and functional profiles of ILC subsets in the peripheral blood (PB) of patients with PCa classified according to their Gleason score. In addition, we analyzed their correlation with PSA values and evaluated the levels of ILC-activating cytokines in the plasma of patients with PCa. We found that ILC2s were increased in patients with PCa, with a trend for a stage-dependent increase, while the antitumoral ILC1s were decreased. Importantly, ILC2s were hyperactivated in patients with PCa, producing higher levels of IL-13 and IL-5. Conversely, ILC1s produced lower levels of the proinflammatory cytokines TNF-α and IFN-γ. Furthermore, we showed that the ILC2-activating mediators IL-33, IL-18, and prostaglandin D2 (PGD2) are increased in the serum of patients with PCa, suggesting that they play a key role in sustaining ILC2 proliferation and activity. Finally, incubation of PBMCs from healthy donors (HDs) with PCa cells, which secrete the ILC2-activating cytokines, recapitulated our observation in patients with PCa, suggesting that PCa cells could act as a potential trigger for ILC2 activity. Additionally, we found that ILC2-derived IL-13 promotes the migration and invasion of PCa cells, with its effects being significantly reduced by an anti-IL-13 blocking antibody. Moreover, the identification by histology and transcriptomics analysis of ILC2s and their activating cytokines in PCa tissue samples further supports their potential role in shaping the immune environment in this type of cancer. Therefore, our study unveils a dysregulation of circulating ILCs in PCa, with a notable increase in ILC2 populations at the expense of ILC1s, identifying these two ILC subsets as potential therapeutic targets for the treatment of PCa.

## Materials and methods

### Human peripheral blood mononuclear cell (PBMC) isolation and serum collection

A total of 48 patients with PCa (16 low-grade (Gleason score 6 and 7 (3 + 4)) and 32 high-grade (Gleason score 7 (4 + 3), 8,9 and 10)) and 25 HDs were enrolled in this study. For serum collection, whole blood was centrifuged at 10,000*g* for 10 min, and the upper phase was collected and immediately frozen. PBMCs were isolated by density gradient centrifugation using Ficoll-Paque (1800 RPM for 20 min at room temperature) and immediately cryopreserved in 90% fetal bovine serum (FBS) and 10% dimethylsulfoxide (DMSO).

### Flow cytometry analysis

Human ILCs were detected and sorted among PBMCs by cytofluorimetric analysis and were negative for classical lineage surface markers and expression of the IL-7 receptor CD127. The lineage markers used were in fluorescein isothiocyanate (FITC), including anti-human CD4 (RPA-T4, SONY, 1:400), anti-human CD15 (HI98, SONY, 1:1.600), anti-human CD16 (3G8, BioLegend, 1:200), anti-human CD20 (REA780, Milteny, 1:400), anti-human CD33 (HIM3-4, SONY, 1:400), anti-human CD34 (561, SONY, 1:800), anti-human CD203C (NP4D6, SONY, 1:100), anti-human FCƐRIα (AER-37 (CRA-1), BioLegend, 1:800), and PERCP 5.5 conjugated including anti-human CD3 (OK73, BioLegend, 1:800) anti-human CD8 (SK1, BioLegend, 1:800), anti-human CD14 (M5E2, BioLegend, 1:100), anti-human CD19 (HIB19, BioLegend, 1:800) while anti-human CD127 antibody was APC-CY7 conjugated (A019D5, SONY, 1:100) or BV421 conjugated (A019D5, Biolegend, 1:100). Additional markers were used to identify ILC subsets: anti-human CD117 (cKit) in APC or BV605 (S18022G, BioLegend, 1:100) and anti-human CRTH2 in PE and PERCP 5.5 (BM16, SONY, 2:50); specifically, ILC1s were identified as a double-negative population, ILC2s as a CRTH2^+^CD117^±^, and ILCPs as a CRTH2^−^CD117^+^ population.

Each sample was firstly labeled with Zombie Green Fixable Viability Kit (BioLegend, San Diego, CA, USA) for 20 min at 4 °C to discriminate dead cells from live cells. After washing with phosphate-buffered saline (PBS), the cell pellets were stained with 50 µL of the extracellular antibody mix prepared in fluorescence-activated cell sorting (FACS) buffer for 20 min in the dark at room temperature, after which the cells were rinsed with PBS and centrifuged. The pellets were resuspended in FACS buffer and analyzed using a BriCyte E6 flow cytometer (Mindray Medical Italy S.r.l., Milan, Italy). For ILC isolation, aliquots of cells were sorted to 98% purity using a Sony MA900 flow cytometer (Sony Biotechnologies, San Jose, CA). The data were analyzed using FlowJo software (TreeStar V.10; Carrboro, NC, USA).

A different panel was used to assess ILC activity by cytofluorimetry. In this case, the lineage was entirely FITC-conjugated including anti-human CD3 (SK7, SONY, 1:1.600), anti-human CD4 (RPA-T4, SONY, 1:400), anti-human CD8 (SK1, SONY, 1:1.600), anti-human CD14 (HCD14, SONY, 1:800), anti-human CD15 (HI98, SONY, 1:1.600), anti-human CD16 (3G8, BioLegend, 1:200), anti-human CD19 (HIB19, SONY, 1:800), anti-human CD20 (REA780, Milteny, 1:400), anti-human CD33 (HIM3-4, SONY, 1:400), anti-human CD34 (561, SONY, 1:800), anti-human CD203C (NP4D6, SONY, 1:100), and anti-human FCƐRIα (AER-37 (CRA-1), BioLegend, 1:800) while anti-human CD127 was likewise in APC-CY7 (A019D5, SONY, 1:100). For phenotypic characterization, the cells were labeled with Zombie Green dye and then with the extracellular antibody mix mentioned above. Next, cells were fixed with FACS-Fix for 30 min in the dark at room temperature and then stained after subsequent washes with 0.1% saponin, with the intracellular antibody mix prepared in the same solution. The antibodies used were anti-human TNF-α in PE (MAb11, SONY, 1:100), anti-human IFN-γ in APC (4S. B3, SONY, 1:50) to detect ILC1 activity, while anti-human IL-13 in PE (7,118,582, BD Pharmingen, 4:50) and anti-human IL-5 in APC (TRFK5, BioLegend, 1:50) were used to evaluate ILC2 cytokine production. The subsequent passages were the same as those described in the previous section.

Human T helper 2 (Th2) cells, myeloid-derived suppressor cells (MDSC), and regulatory T (Treg) cells were identified using the following antibodies: PE anti‐human CD25 (BC96, Biolegend, 1:100), PE/Dazzle anti-human CD33 (WM53, Biolegend, 1:100), PE/Cyanine7 anti-human CD183 (CXCR3) (REA113, Miltenyi, 1:200), BV510 anti-human CD3 (SK7, SONY, 1:100), APC anti-human HLA-DR (L243, Biolegend, 1:100), A700 anti-human CD4 (A161A1, Biolegend, 1:100), and APC-CY7 anti-human CD45RO (UCHL1, Biolegend, 1:100). Th2 cells were identified as CD3^+^CD4^+^CD45RO^+^CRTH2^+^CXCR3^−^, Treg cells as CD25^+^CD127^low^, and MDSC cells as CD33^+^HLA-DR^−^.

### Quantitative real-time PCR (qPCR)

Total RNA was isolated from freshly sorted human ILC2s and PC3 cells using TRIzol reagent according to the manufacturer’s instructions (Invitrogen). The final preparation of RNA was considered DNA and protein free if the ratio of readings at 260/280 nm was ≥ 1.7. Isolated mRNA was reverse-transcribed by iScript Reverse Transcription Supermix for RT-qPCR (Bio- Rad, Milan, Italy). Quantitative real-time PCR was carried out with a Bio-Rad CFX384 real-time PCR detection system (Bio-Rad, Milan, Italy) with specific primers (hIL1RL1 5′-AGAAATCGTGTGTTTGCCTCA-3′, 5′-TCCAGTCCTATTGAATGTGGGA-3′; hIL18R1 5′-TTGGTGAGAAAAGCAGACATGG-3′, 5′-TCACTAGGCACACTACTGCCA-3′; hMMP9 5′-GGGACGCAGACATCGTCATC-3′, 5′ TCGTCATCGTCGAAATGGGC-3′; hMMP2 5′-TACAGGATCATTGGCTACACACC-3′, 5′-GGTCACATCGCTCCAGACT-3′) using SsoAdvanced Universal SYBR Green Supermix (Biorad). Samples were simultaneously amplified in triplicate in one-assay run with a nontemplate control blank for each primer pair to control for contamination or for primer dimerization, and the Ct value for each experimental group was determined. The ribosomal protein S18 was used as an internal control to normalize the Ct values, using the 2^−ΔCt^ formula.

### Serum cytokine quantification

Serum cytokine levels were measured using BioLegend LEGENDplex™ bead-based immunoassays. Specifically, for TSLP, GM-CSF, IL-23, IL-15, IL-18, IL-11, and IL-33, the Human Cytokine Panel 2 (13-Plex) was used, while for IL-5, IL-13, IL-9, IL-4, and Th, cytokine panels (12-plex) were applied. Analyses were performed according to the manufacturer’s instructions.

### Cell culture

PC3 and DU145 cell lines were purchased from the American Type Culture Collection (ATCC) and cultured in Roswell Park Memorial Institute (RPMI) 1640 medium supplemented with 10% heat-inactivated FBS, 2 mmol/L l-glutamine, 100 U/ml penicillin, 100 µg/ml streptomycin, and 10 mM 4-(2-hydroxyethyl)-1-piperazineethanesulfonic acid (HEPES) buffer (all from Gibco; New York, NY, USA) at 37 °C in a humidified incubator under 5% CO_2_. For ILC2 expansion, freshly sorted ILC2s were cultured for 2 weeks in supplemented RPMI 1640 with a mixture of IL-2 (100 U/ml) and IL-7 (5 ng/ml) and by adding PHA (1 μg/ml) at day 0. Medium was replaced every 2–3 days, and cell phenotype was verified by flow cytometry. For the preparation of ILC2 CM, expanded ILC2s were incubated with IL-33 (50 ng/ml, Adipogen) and IL-25 (50 ng/ml, Biolegend) for 48 h.

### Preparation of PC3 and DU145 cells conditioned medium

PC3 and DU145 were cultured in RPMI 1640 complete medium in a 100-mm-diameter dish. The medium was collected after 48 h, centrifuged at 1800 rpm for 10 min, and filtered through a 0.22-mm syringe filter.

### In vitro experiments

PBMCs from HDs (1 × 10^6^/ml) were seeded into six-well plates and cultured in complete RPMI 1640 medium alone or in the presence of 50% v/v of PC3 or DU145 conditioned medium (CM) for 24 h. Next, cells were stimulated with 1 μg/ml PMA plus 0.5 μg/ml Ionomycin, in the presence of BrefeldinA (all from Sigma-Aldrich) for 3 h prior to intracellular staining for the evaluation of cytokine production. Where indicated, HpARI (1 μg/ml, Adipogen) was added.

### Wound healing assay

PC3 cells were seeded in 24-well plates (3 × 10^5^ cells/well). Once the cells reached 90% confluence, ILC2 CM (30% v/v) with or without anti-IL-13 blocking antibody (10 μg/ml, Biolegend) were added and a wound area was carefully created by scraping the cell monolayer with a sterile 200-μl pipette tip. Subsequently, the cells were incubated at 37 °C in 5% CO_2_. The width of the wounded area was monitored and photographed with an inverted microscope at various time points (20-fold magnification). The wounded area was measured using ImageJ software (LASV3.8, Germany).

### Clonogenic assay

PC3 cells (1 × 10^3^ cells/well) were seeded in six-well plates with ILC2 CM (30% v/v) with or without anti-IL-13 blocking antibody. Cells were cultured for 14 days to allow colonies to form. Formed colonies were washed twice with 1× PBS, fixed with 4% paraformaldehyde, and stained with 0.5% Crystal Violet, and colonies were counted manually. Images of the colonies were obtained using a digital camera. The experiments were done at least three times, in duplicate.

### ILC2 signature in TCGA data

We investigated the association between an ILC2 gene signature and survival of patients with PCa using The Cancer Genome Atlas (TCGA) data. The ILC2 gene signature was compiled from Ref. [32] and included 66 genes also detected in the PCa TCGA data. Tumor gene expression and clinical data of patients with PCa were accessed using the TCGAbiolinks package (version 2.25.3) for R (version 4.2.2) [33]. Raw counts were filtered to remove lowly expressed genes, retaining genes (*n* = 25,561) that were expressed at a minimum of 1 count per million (cpm) in at least one patient. We also removed patients that had missing survival information, retaining 495 patients. Raw counts were then converted to log_2_(cpm + 1) using the voom function of the limma package (version 3.54.2) [34]. An ILC2 signature risk score per patient was calculated using multivariate Cox regression analysis as described in Chu et al. [35]. Patients were stratified according to the median risk score across all patients. If a patient had lower risk score than the overall median, they were classified as having a “low” risk score, and if the risk score was above the median, they were classified as having a “high” risk score. Difference in survival between the high-risk and low-risk groups was estimated using a likelihood ratio (LR) test. The *p*-value was then reported on the Kaplan–Meier survival curve using the survminer package (version 0.4.9).

### Immunohistochemistry (IHC) of human prostate tissues

Human prostate tissues were fixed in 4% formaldehyde and embedded in paraffin. Staining reactions were performed by automated staining using a BOND RX autostainer (Leica Biosystems) by the Translational Research Unit of the Institute of Tissue Medicine and Pathology of the University of Bern. For double immunohistochemistry staining, sections were first deparaffinized and antigen was retrieved using Tris–ethylenediaminetetraacetic acid (EDTA) solution (pH 9.0) for 30 min (for IL-33 antigen) or 10 min (for GATA3 antigen) at 95 °C. No pretreatment was applied for CD3 antigen. Sections were then stained with goat anti-human IL-33 (R&D Systems, polyclonal, # AF3625; dilution 1:400) and mouse anti-human CD3 (Diagnostics Biosystems, clone LN10, #NCL-L-CD3-565; dilution 1:200) or goat anti-human IL-33 and mouse anti-human GATA3 (Cell Marque, clone L50-823, #390 M-14; dilution 1:200) primary antibodies. A rabbit-anti-goat IgG antibody (Abcam, polyclonal, # ab6697; dilution 1:2000) was used as a secondary antibody to detect goat anti-human IL-33 antibody. Specific binding of mouse and rabbit antibodies was visualized using a polymer-based visualization system with horseradish peroxidase as the enzyme and 3,3-diaminobenzidine (DAB) as a brown chromogen (BOND Polymer Refine Detection; #DS9800) or an alkaline phosphatase-linked polymer and Fast Red as red chromogen (Bond Polymer Refine and Red Detection; #DS9390) (all from Leica Biosystems). The samples were counterstained with hematoxylin and mounted with Pertex (Biosystems). Slides were scanned at high resolution using a Nanozoomer S360 digital slide scanner (Hamamatsu) at 20× magnification. The resulting files were converted from the original NDPi format to a MRXS format for downstream analysis.

### Single-cell RNA sequencing data

Single-cell RNA sequencing (scRNA-seq) data from freshly collected PCa samples were obtained from a recently published study [36] and processed using Trailmaker single-cell data analysis software (Parse Biosciences). Data processing was performed following the standard workflow provided by the software. Sample integration was carried out using the Seurat analysis tool combined with the Harmony method, with the number of highly variable genes (HVGs) set to 2000. For visualization, the uniform manifold approximation and projection (UMAP) method was employed, with the minimum distance parameter set to 0.3.

### Datasets

The correlation between IL-33 and IL-18 in samples from patients with PCa in the TGCA (PRAD) was analyzed using the TIMER database (https://cistrome.shinyapps.io/timer/). To investigate the prognostic impact of IL-13 expression level on Pca patient survival, we performed survival analysis in the TCGA PRAD cohort using the GEPIA2 database (http://gepia2.cancer-pku.cn/#index). By using the GEDS platform (http://bioinfo.life.hust.edu.cn/web/GEDS/), we examined IL-33 and IL-18 expressions in eight prostate cancer cell lines. Datasets used by TIMER and GEPIA2 are mainly based on PRAD-TCGA data, while datasets used by GEDS come from the Cancer Cell Line Encyclopedia (CCLE). These datasets are publicly available.

### Statistical analysis

GraphPad Prism 9 software was used for statistical analyses. Paired or unpaired *t*-tests were used when comparing two groups. Analyses of variables (ANOVAs) or the nonparametric Kruskal–Wallis test were used for comparison of multiple groups. Data in graphs represent the mean ± standard error on the mean (SEM), with *p* value < 0.05 (two-tailed) being significant and labeled with *, **, and *** for *p* values < 0.01, < 0.001 or < 0.0001, respectively.

## Results

### ILCs are dysregulated in patients with PCa

**Fig. 1:**
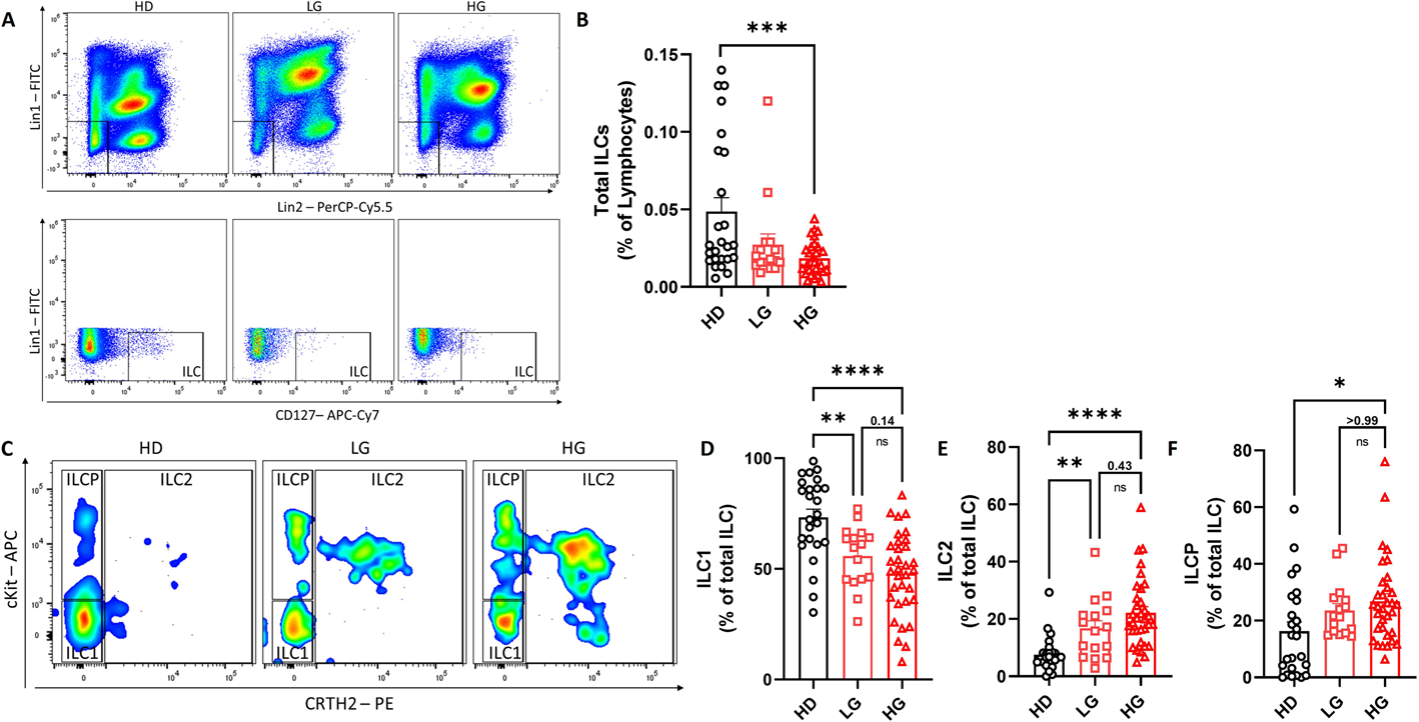
ILCs are dysregulated in patients with PCa. **A**, **C** Representative examples of flow cytometry analysis of total ILCs (**A**) and ILC subsets (**C**) in PBMCs of HDs (*n* = 25) and patients with LG (*n* = 16) and HG (*n* = 32) PCa. **B**–**F** Frequency of total ILCs (**B**), ILC1s (**D**), ILC2s (**E**), and ILCPs (**F**). Data shown as mean ± SEM (*p < 0.05; **p < 0.01; ***p < 0.001; ****p < 0.0001) and analyzed by Wilcoxon and/or one-way ANOVA tests

To evaluate the putative involvement of ILCs in PCa, we used PB samples from HDs and patients with PCa, divided into low-grade and high-grade groups (LG and HG, respectively) according to the Gleason score. In particular, patients with Gleason scores of G6 and G7 (3 + 4) were included in the LG group, while patients with Gleason scores of G7 (4 + 3), G8, G9, and G10 were included in the HG group, as summarized in Table 1 and Supplementary Fig. 1A (while Supplementary Table 1 includes information about age and serum PSA levels from HDs). First, we evaluated the frequency of total ILCs identified as lineage^−^CD127^+^ cells within the live lymphocyte population in peripheral blood using flow cytometry analysis (Fig. 1A). We found a significant reduction in the frequency of total ILCs in patients with HG PCa compared with HDs (Fig. 1B). Next, the ILC subsets were analyzed according to the expression of CRTH2 and CD117. Specifically, ILC1s were identified as a double-negative population, ILC2s as a CRTH2^+^CD117^±^, and ILCPs as a CRTH2^−^CD117^+^ population (Fig. 1C). The frequency of ILC subsets in both patients with LG and HG PCa was dysregulated compared with HDs. In particular, ILC1s were decreased while ILC2s and ILCPs increased in both LG and HG PCa groups (Fig. 1D–F). Consistent with the differences observed in frequency, the absolute numbers of total ILCs and ILC subsets were significantly altered in patients with PCa compared with healthy controls (Supplementary Fig. 1B, C). In addition, we extended our analysis to include Th2 cells, which represent the adaptive counterpart of ILC2s, as well as MDSCs and Tregs, which can be induced by ILC2s [28, 30]. As shown in Supplementary Fig. 1D–I, the frequency of Th2 cells was significantly increased in patients with HG PCa, mirroring the findings observed for ILC2s. In contrast, no significant changes were detected in the frequency of Tregs, while MDSCs were markedly increased. These findings suggest that, in patients with PCa, the reduced frequency of ILC1s may be indicative of a compromised antitumoral response, whereas an increase in ILC2s could be associated with cancer progression, in line with similar observations in other types of cancer [37, 38].

**Table 1:**
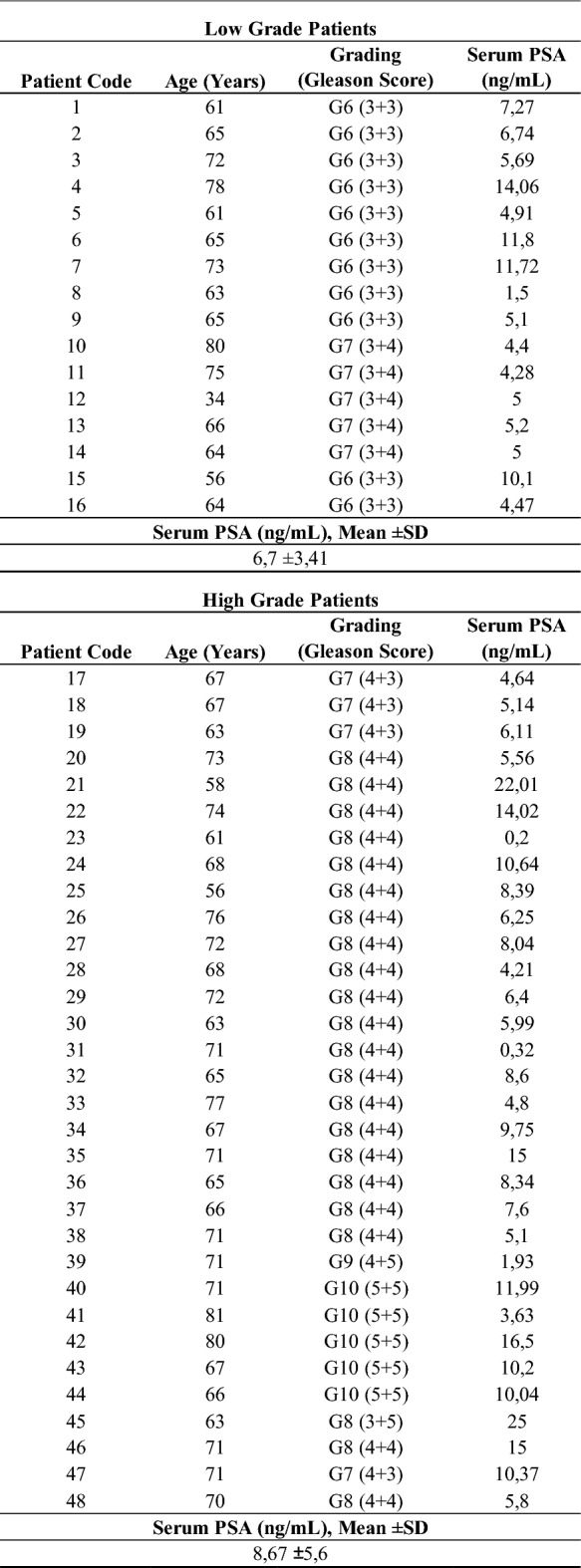
Clinical characteristics of the study population

### ILC2 frequency increases in patients with high PSA values and impacts patients’ survival

**Fig. 2:**
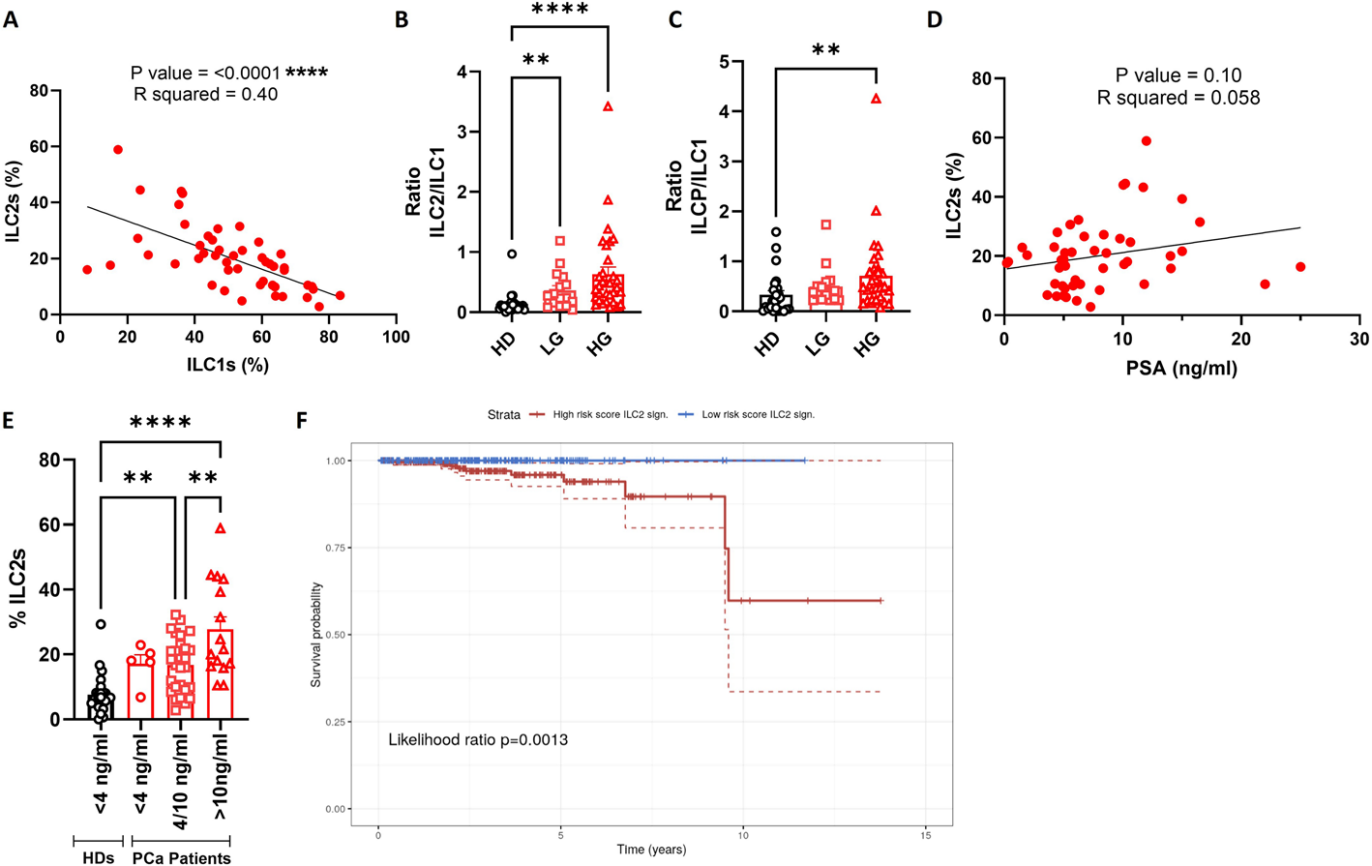
ILC2 frequency increases in patients with high PSA values and impacts patients’ survival. **A** Correlation of ILC1 and ILC2 frequencies in patients with PCa (*n* = 48). **B**, **C** Ratios of ILC2/ILC1 (**B**) and ILCP/ILC1 (**C**) in HDs and patients with LG and HG PCa . **D** Correlations between circulating ILC2 frequency and PSA value (*n* = 48). (**E**) Frequency of ILC2s based on PSA levels of HDs (< 4 (*n* = 25)) and patients with PCa (< 4 (*n* = 5), 4–10 (*n* = 28) and > 10 ng/ml (*n* = 15) according to the National Comprehensive Cancer Network guidelines. (**F**) Survival analysis (likelihood ratio test) of patients with PCa (TCGA), stratified according to their risk score for an ILC2 gene signature. Survival of patients was higher in low-risk score patients (blue line) compared with high-risk patients (red line). Data shown as mean ± SEM (*p < 0.05 **p < 0.01; ****p < 0.0001) and analyzed by Wilcoxon and/or one-way ANOVA tests

As described above, ILC2s play a protumoral role in different types of cancer, such as colorectal cancer (CRC) and bladder cancer as well as chronic myeloid (CML) and acute promyelocytic leukemia (APL), suggesting their potential involvement in the context of PCa [16, 31, 39]. Conversely, ILC1s exert protective immunity; however their activity can be impaired in certain cancer types, such as melanoma, CRC, breast cancer, and acute myeloid leukemia (AML), owing to immune exhaustion driven by tumor-promoting signals within the microenvironment [20]. ILCs are highly adaptable cells, capable of changing their phenotype and function in response to signals from the tissue microenvironment. This plasticity allows them to transdifferentiate into other ILC subsets under both homeostatic and pathological or inflammatory conditions, thereby shaping different immune responses [40, 41]. Therefore, we first investigated whether there was a correlation between the frequency of ILC1s and ILC2s in patients with PCa. As expected, we found an inverse correlation between the frequency of ILC1s and ILC2s (Fig. 2A). In addition, we explored the balance between each ILC population in both patients with LG and HG PCa compared with HDs. The ratios of ILC2/ILC1 and ILCP/ILC1 were markedly increased in both LG and HG groups, or only in HG, respectively (Fig. 2B and C), while the ratio of ILCP/ILC2 in both LG and HG did not differ (Supplementary Fig. 2A). Next, we focused on PSA levels and evaluated the correlation with ILC populations. As shown in Fig. 2D, ILC2 frequency tended to correlate with PSA values in patients with PCa, while ILC1 and ILCP frequency did not correlate with PSA values (Supplementary Fig. 2B, C). In addition, no significant correlations were found between ILC2/ILC1 and ILCP/ILC1 ratios with PSA values (Supplementary Fig. 2D, E). Interestingly, when patients with PCa were stratified based on PSA levels (< 4, 4–10 and > 10 ng/ml) according to the guidelines of the National Comprehensive Cancer Network [42, 43], the frequency of ILC2s was significantly increased in patients with PSA values > 10 ng/ml (typically followed by prostate biopsy) compared with HDs with PSA values lower than 4 ng/ml and patients with PCa with PSA values between 4 and 10 ng/ml (Fig. 2E), while no changes were observed for ILC1s and ILCPs (Supplementary Fig. 2F, G). In addition, we analyzed the potential relevance of ILC2s in the survival of patients with PCa by applying an ILC2 gene signature previously generated by others to the TCGA PCa dataset [32]. We found that patients with a higher ILC2 gene signature showed reduced overall survival compared with patients with low ILC2 gene signature (Fig. 2F). Collectively, our results suggest that the frequency of peripheral ILC2s may represent a promising cellular predictor, associated with the PSA value, for defining the risk category of patients with PCa at diagnosis. Moreover, our TCGA analysis highlighted ILC2s as a promising target in PCa progression, shedding light on novel strategies to fight this type of cancer.

### ILC activation is affected in patients with PCa

**Fig. 3:**
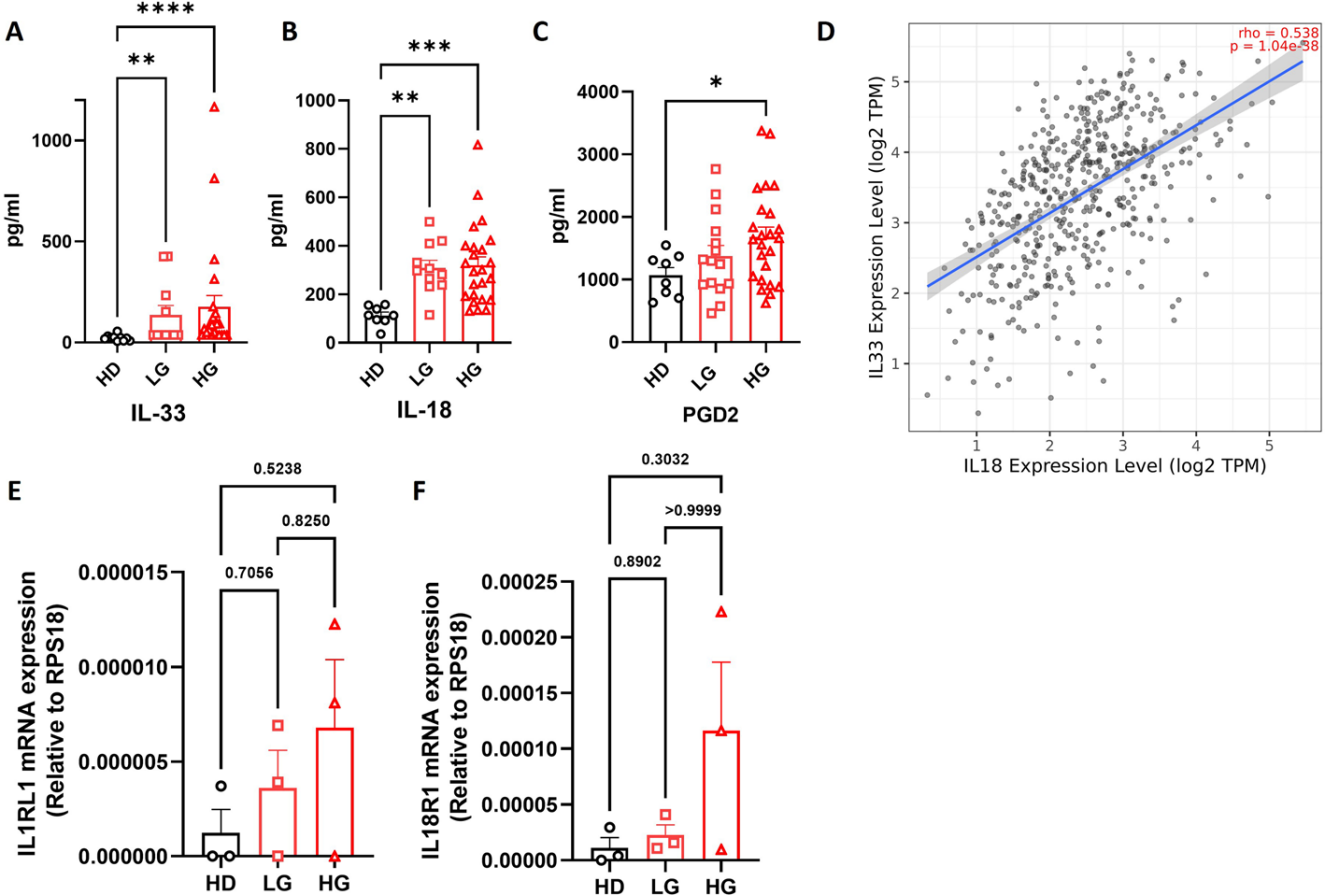
**A**–**C** ILC activation is affected in patients with PCa. IL-33 (**A**), IL-18 (**B**), and PGD2 (**C**) concentrations (pg/ml) in sera of HDs (*n* = 8) and patients with LG (*n* = 11–15) and HG (*n* = 24) PCa. **D**. Correlation between IL-33 and IL-18 levels in PCa via TIMER database. **E**, **F** Expression of IL1RL1 (**E**) and IL18R1 (**F**) assessed by qPCR in freshly sorted ILC2s from HDs and patients with LG and HG PCa (*n* = 3). Data shown as mean ± SEM (*p < 0.05; **p < 0.01; ***p < 0.001; ****p < 0.0001) and analyzed by Wilcoxon and/or one-way ANOVA tests

ILC2s have the capability to react with various soluble mediators, including alarmins (IL-25, IL-33, and thymic stromal lymphopoietin (TSLP)), survival cytokines (such as IL-2, IL-9, IL-7, and IL-18), and eicosanoids such as PGD2 [44]. Among these, IL-33 and IL-18 play key roles in tumor biology and immunology in different types of cancers, including breast, CRC, and CML [16, 39, 45–47]. Therefore, we evaluated the circulating levels of these ILC2-activating cytokines in the serum of HDs and patients with PCa using multiplexed cytokine and enzyme-linked immunosorbent assay (ELISA) assays. As shown in Fig. 3A–C, the expression of IL-33, IL-18, and PGD2 was significantly increased in both patients with LG and HG PCa compared with the control group. In parallel, no differences were observed for the ILC1-activating cytokines (IL-15 and IL-12) between healthy donors and patients with PCa (Supplement Fig. 3A, B). Next, we investigated the relationship between IL-33 and IL-18 in PCa (referred to as PRAD) using the TIMER database [48]. Interestingly, we noted a significant correlation between IL-33 and IL-18 levels in PRAD patients (Fig. 3D). To further explore the role of these cytokines, we assessed the expression of their receptors, IL1RL1 (ST2) and IL18R1, on freshly sorted ILC2s from HDs and patients with PCa. As shown in Fig. 3E, F, we observed a trend for increased expression of both IL1RL1 and IL18R1 in ILC2s from patients with HG PCa compared with HDs, although this observation was based on a limited number of samples (*n* = 3 per group) and did not reach statistical significance. These results suggest that the frequency of ILC2s in patients with PCa may be associated with higher levels of ILC2-activating cytokines and indicate that both IL-33 and IL-18 could potentially play a role in PCa progression.

### ILC function is altered in patients with PCa

**Fig. 4:**
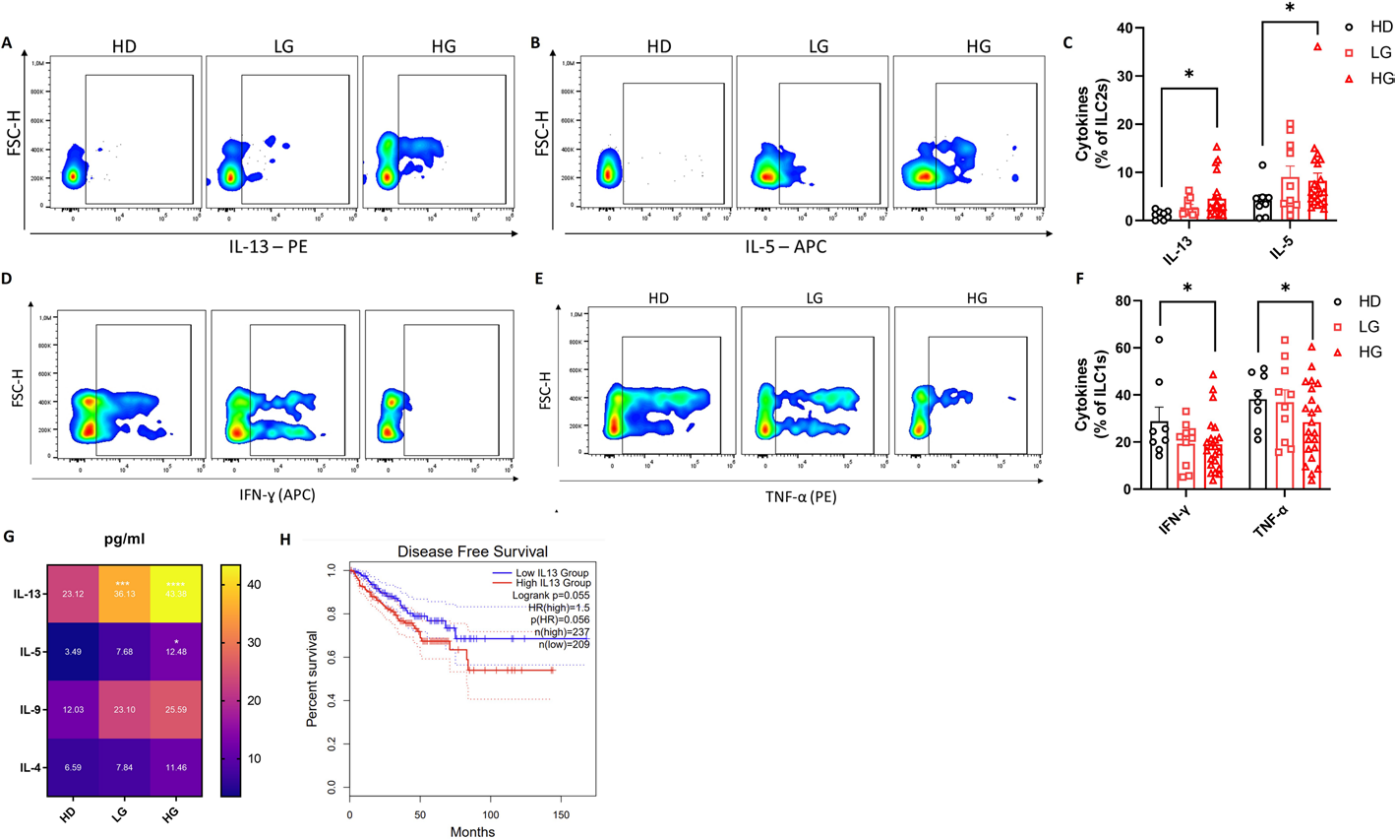
ILC function is altered in patients with PCa. **A**, **B**. Representative example of flow cytometry analysis of ILC2-produced IL-13 (**A**) and IL-5 (**B**) in ex vivo PBMCs upon stimulation. **C** Frequencies of IL-13 and IL-5 positive ILC2s in ex vivo PBMCs from HDs (*n* = 8) and patients with LG (*n* = 10) and HG (*n* = 22) PCa upon stimulation. **D**, **E** Representative example of flow cytometry analysis of ILC1-produced TNF-α (**D**) and IFN-γ (**E**) in ex vivo PBMCs upon stimulation. **F** Frequencies of IFN-γ- and TNF-α-positive ILC1s in ex vivo PBMCs from HDs (*n* = 8) and patients with LG (*n* = 10) and HG (*n* = 22) PCa upon stimulation. **G** Quantification of type 2 cytokines assessed by Legendplex^™^ analysis in sera of HDs (*n* = 12) and patients with LG (*n* = 13) and HG (*n* = 29) PCa. **H** Disease-free survival of patients with PCa stratified on the basis of high (red) and low (blue) IL-13 expression. Data shown as mean ± SEM (*p < 0.05; ***p < 0.001; ****p < 0.0001) and analyzed by Wilcoxon and/or one-way ANOVA tests

Next, we assessed the functionality of ILCs by evaluating the production of both type 1 and type 2 cytokines in circulating ILC1s and ILC2s from patients with PCa and HDs after ex vivo stimulation (gating strategy shown in Supplementary Fig. 4A). ILC2s from patients with HG PCa produced more IL-13 and IL-5 compared with ILC2s from HDs (Fig. 4A–C). In addition, we observed that the production of both IFN-γ and TNF-α was reduced in ILC1s from patients with HG PCa (Fig. 4D–F). Interestingly, we noted that the IL-13-producing ILC2s displayed higher FSC value, probably due to the hyperactivation that can lead to an increase in cell size and/or granularity [49]. In line with these findings, we observed elevated levels of both IL-13 and IL-5 in the serum of patients with PCa, as well as other ILC2-prototypic type 2 cytokines, such as IL-9 and IL-4, as compared with HDs, while no significant differences were observed for IFN-γ and TNF-α (Fig. 4G; Supplementary Fig. 4B, C). IL-13 has been described as a potential predictive biomarker for cancer treatment, promoting tumor cell proliferation, invasion, and metastasis development [50–52]. Importantly, the alpha2 chain of the IL-13 receptor (referred to as IL-13Rα2) is overexpressed in different solid tumors and correlates with poor prognosis in glioblastoma, colorectal cancer, adrenocortical carcinoma, pancreatic cancer, and breast cancer [53]. Therefore, we used data from TCGA via GEPIA2 to investigate the impact of IL-13 expression on the survival of patients with PCa [54]. In line with other types of cancer, poor disease-free survival (DFS) was linked to upregulated IL-13 expression in patients with PCa (Fig. 4H). These results suggest that ILC2s are hyperactivated in patients with PCa by producing high levels of IL-13, which, in turn, may influence the survival of patients with PCa. Conversely, ILC1s are hypoactive, producing lower levels of both IFN-γ and TNF-α. Nevertheless, further studies are needed to elucidate the mechanisms underlying this impaired ILC1 functionality in the context of PCa.

### PCa cell conditioned medium (CM) recapitulates observations in patients

**Fig. 5:**
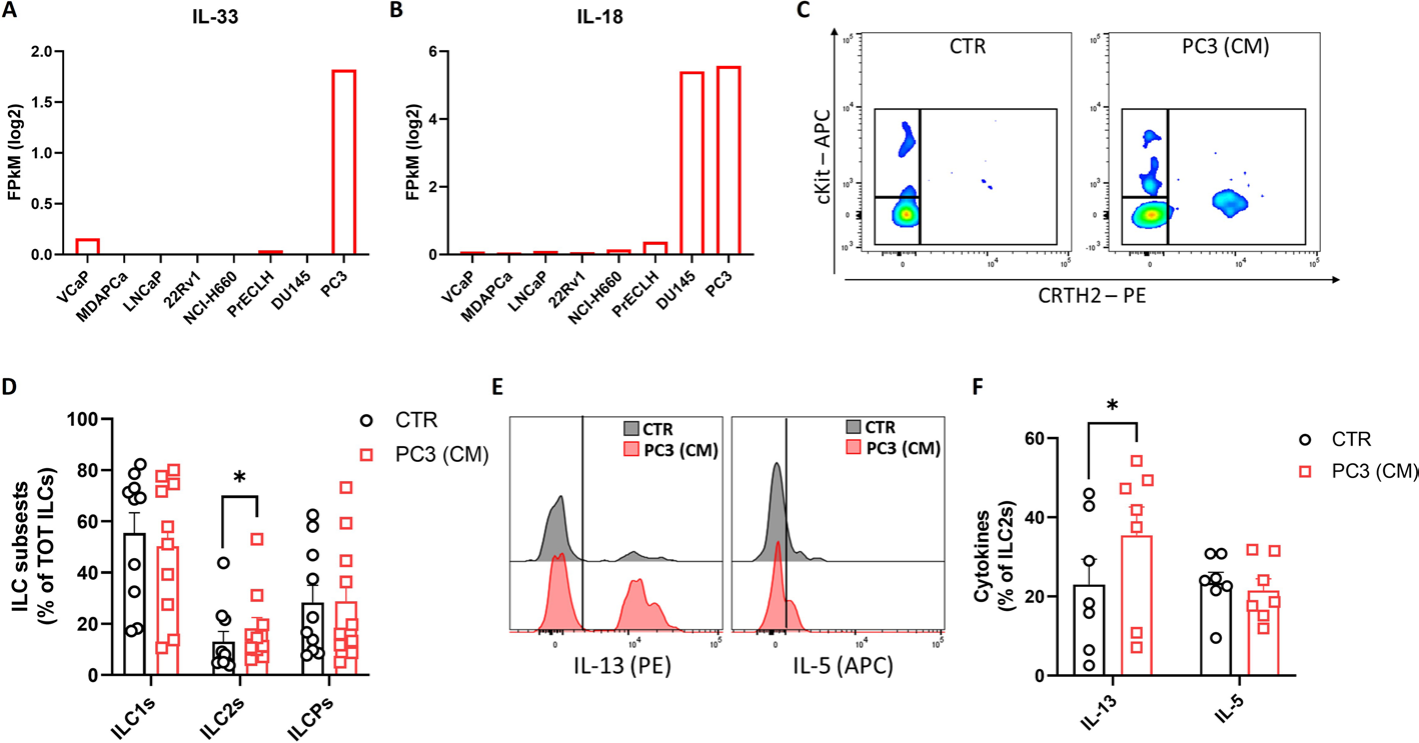
PCa cell conditioned medium (CM) recapitulates observations in patients. **A**, **B** Prostate cancer cell line mRNA expression of IL-33 (**A**) and IL-18 (**B**) via GEDS platform. **C** Representative example of flow cytometry analysis of ILC subsets in HD PBMCs cultivated with or without PC3 conditioned media (CM). **D** Frequency of ILC subsets in HD PBMCs (*n* = 10) incubated with or without PC3 CM. **E** Representative example of flow cytometry analysis of IL-13- and IL-5-positive cell populations in HD PBMCs incubated with or without PC3 conditioned media. **F** Frequencies of IL-13- and IL-5-positive ILC2s in ex vivo PBMCs upon incubation with or without PC3 conditioned media (*n* = 7). Data shown as mean ± SEM (*p < 0.05 versus CTR) and analyzed by Wilcoxon and/or one-way ANOVA tests

As described above, IL-33 and IL-18 have been identified as the signalling mediators involved in cancer development and progression. Therefore, we hypothesized that tumor-derived IL-33 and IL-18 might influence ILC2 frequency and function in PCa. First, we interrogated the GEDS platform to evaluate the expression of both IL-33 and IL-18 in different PCa cell lines [55]. Among these, the PC3 PCa cell line showed the highest expression of both IL-33 and IL-18 (Fig. 5A, B). In addition, it has also been demonstrated that PC3 cancer cells were able to secrete high levels of PGD2 in culture medium [31]. Therefore, we investigated the impact of PC3 CM on PBMCs from HDs by evaluating ILC2 frequency and function by flow cytometry. As shown in Fig. 5C, D, exposure of HD PBMCs to PC3 CM increased the frequency of ILC2s. In addition, PC3 CM significantly increased IL-13 production (Fig. 5E, F). To support this result, we used the helminth parasite-secreted protein (HpARI), known to suppress type 2 immune responses through interference with the IL-33 pathway [56]. As shown in Supplementary Fig. 5A, the addition of HpARI significantly reduced the production of IL-13 by ILC2s, after incubation with PC3 CM, supporting a potential role of IL-33 in mediating the observed effects. In addition, we used the DU145 PCa cell line with less metastatic potential compared with PC3 cells [57]. As shown in Supplementary Fig. 5B–E, exposure to DU145 CM did not induce significant increase of ILC2s nor production of either IL-13 or IL-5 in HD ILC2s. These results suggest that PCa cells are involved in the dysregulation of ILC2 frequency and activity and that this effect is, at least in part, mediated by IL-33.

### ILC2-derived IL-13 promotes PCa cell migration and invasion

**Fig. 6:**
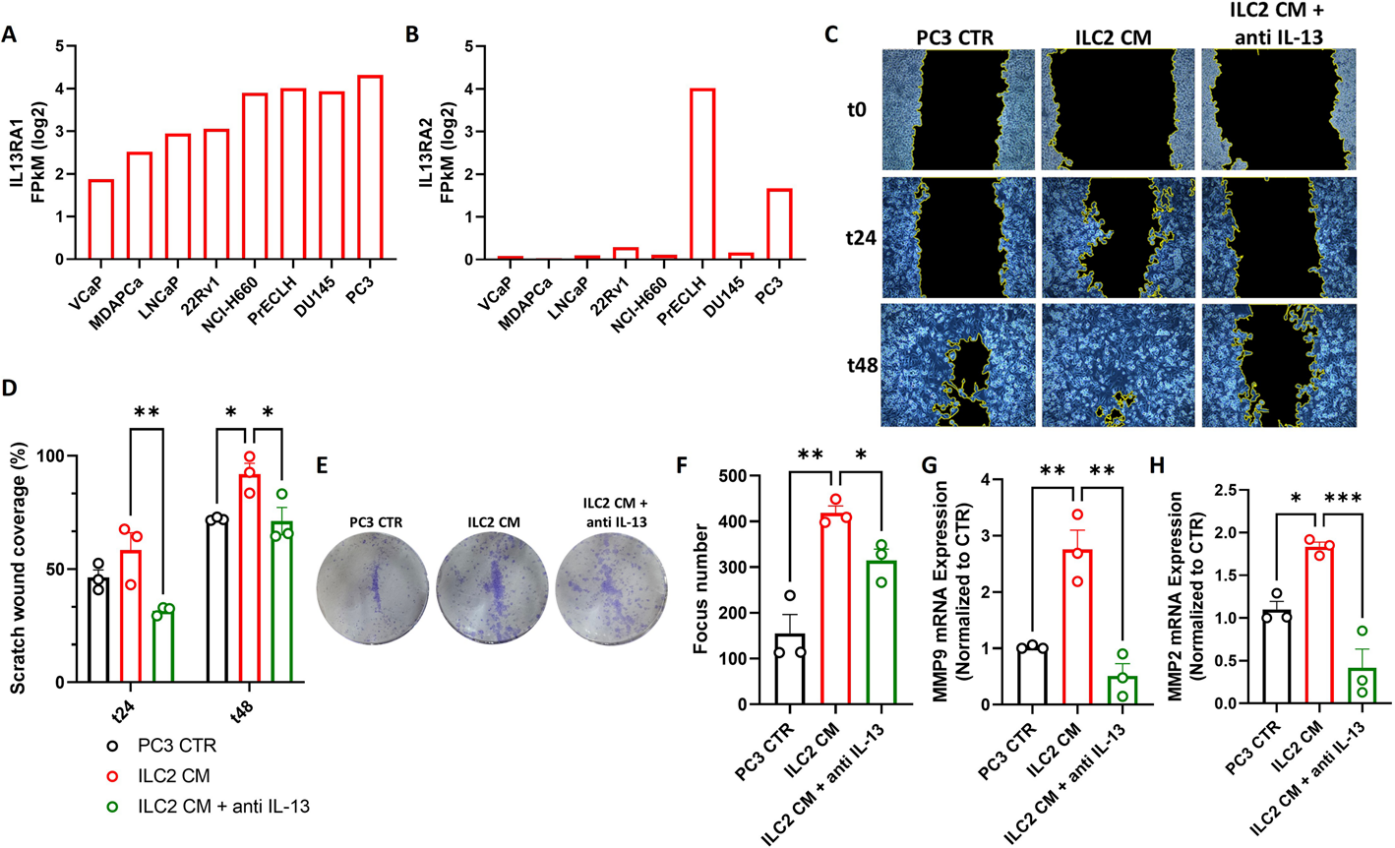
ILC2-derived IL-13 promotes PCa cell migration and invasion. **A**, **B** Prostate cancer cell line mRNA expression of IL13RA1 (**A**) and IL13RA2 (**B**) receptors via GEDS platform. **C** Representative example of a wound healing assay (20× magnification) performed using PC3 cancer cells after incubation with ILC2 CM or ILC2 CM + anti-IL-13 blocking antibody. **D** Quantification of the healed wound area at 24 and 48 h. **E**, **F** Representative example (**E**) and quantification (**F**) of clonogenic assays performed using PC3 cancer cells after incubation with ILC2 CM or ILC2 CM + anti-IL-13 blocking antibody for 10 days. **G**, **H** Expression of MMP9 (**G**) and MMP2 (**H**) assessed by qPCR analysis in PC3 cancer cells upon incubation with ILC2 CM or ILC2 CM + anti-IL-13 blocking antibody. Data shown as mean ± SEM (*p < 0.05; **p < 0.01; ***p < 0.001) and analyzed by Wilcoxon and/or one-way ANOVA tests

Over the past few years, IL-13 and its receptors have been identified as promising targets for cancer therapy, with the inhibition of IL-13-producing cells being investigated as a potential therapeutic strategy [53, 58]. Notably, studies using the transgenic adenocarcinoma of the mouse prostate (TRAMP) model have demonstrated that IL-13 plays a role in prostate tumorigenesis [59]. Furthermore, IL-13 has been shown to promote the proliferation of various PCa cell lines through the activation of both IL13RA1 and IL13RA2 receptors [60]. In this context, we investigated the potential role of ILC2-derived IL-13 in promoting the migration and invasion of PCa cells. Initially, using the GEDs platform, we analyzed the expression levels of IL13RA1 and IL13RA2 across different PCa cell lines. Consistent with previous findings [61], PC3 cells exhibited high expression of both receptors, supporting the hypothesis of a functional impact of ILC2-derived IL-13 on these cells (Fig. 6A, B). Next, we performed wound healing and clonogenic assays using CM of activated ILC2s. We found that the presence of ILC2 CM increased the migration and colony formation of PC3 cells. Conversely, the addition of an anti-IL-13 blocking antibody reduced wound coverage and colony development in PC3 cells, confirming that ILC2-derived IL-13 has an impact on PCa cell migration and invasion (Fig. 6C–F). To further validate the protumoral effects of ILC2 CM on PCa migration and invasion, we examined the expression of MMP9 and MMP2 in PC3 cells. These molecules are strongly associated with the epithelial-to-mesenchymal transition (EMT) process in various cancers, including prostate cancer [62–64]. Our results showed that the addition of ILC2 CM significantly increased the expression of these EMT markers in PC3 cells, while the use of an anti-IL-13 blocking antibody reduced their expression to levels below the baseline (Fig. 5G, H). Taken together, our findings suggest crosstalk between cancer cells and ILC2s, in which cancer cells stimulate ILC2s to produce IL-13, which in turn enhances their migratory and invasive properties.

### ILC2s are enriched in PCa tissues

**Fig. 7:**
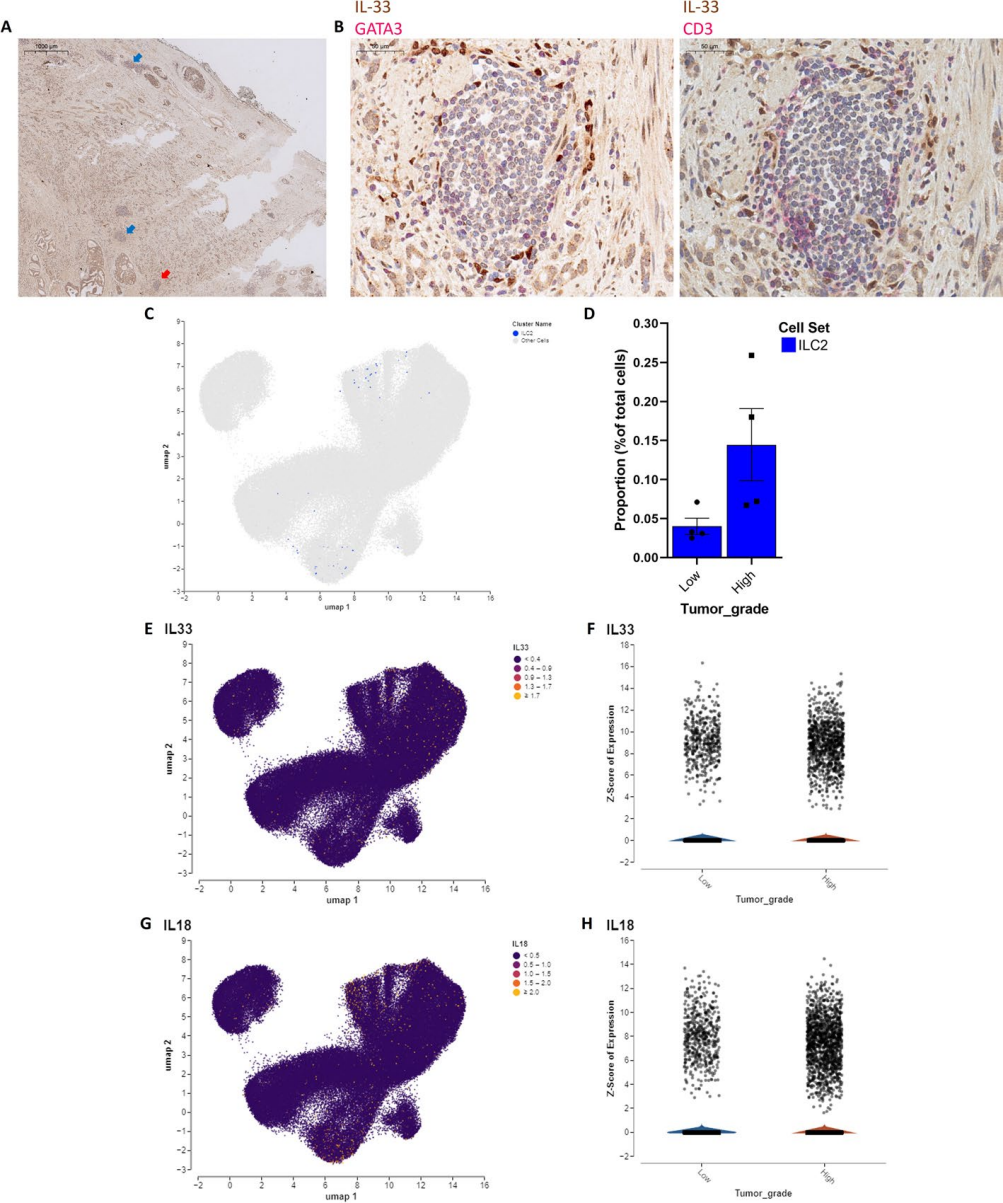
ILC2s are enriched in PCa tissues. **A** Consecutive sections of HG PCa tissue were double stained for IL-33 and GATA3 (representative samples depicted) or for IL-33 and CD3. Arrows indicate tumor areas with immune foci containing ILC2, recognized as GATA3^+^ CD3^−^ lymphocytes. Scale bar: 1000 μm. **B** Magnified view of the area marked by the red arrow showing the indicated double stainings on consecutive sections. GATA3^+^ CD3^−^ lymphocytes are visible in the center of the immune foci. Scale bar: 50 μm. **C**–**H** Analysis of single-cell RNA sequencing (scRNA-seq) data from a publicly available PCa dataset. **C** UMAP plot highlighting ILC2, defined as PTPRC^+^GATA3^+^ CD3^−^ (blue dots). **D** Comparison of the abundance (% of total cells) of ILC2s in high-grade (HG) versus low-grade (LG) PCa tissues. **E** UMAP plot showing IL33-expressing cells, with a blue–yellow scale indicating IL-33 RNA expression levels. **F** Quantification of IL-33-expressing cells in HG and LG PCa tissues. **G** UMAP plot showing IL-18-expressing cells, with a blue–yellow scale indicating IL-18 RNA expression levels. **H** Quantification of IL18-expressing cells in HG and LG PCa tissues

To translate our finding from circulating ILC2s to tissue-resident ILC2s, we performed immunohistochemistry (IHC) analysis on tissue sections of patients with HG PCa. IHC indicated the presence of ILC2s, identified as GATA3^+^ CD3^−^ lymphocytes among foci of CD3^+^ lymphocytes, which are next to epithelial cells and next to regions with cells expressing IL-33 (Fig. 7A, B). This result was in line with our observations in circulating ILC2s. In addition, we explored a recently published single-cell RNA sequencing (scRNA-seq) dataset of freshly collected PCa tissues [36]. By analyzing the scRNA-seq data, we confirmed the presence of ILC2s (identified as PTPRC^+^, CD3^−^, GATA3^+^) and found an increase in the percentage of ILC2s in HG compared with LG PCa tissues (Fig. 7C, D). Furthermore, we observed an enrichment of the cytokine mediators IL-33 and IL-18 in HG PCa tissues, consistent with our findings in circulating cytokines (Fig. 7E–H). These results suggest that ILC2s and their activating cytokines are potentially enriched in patients with PCa also at tissue level, supporting the hypothesis that a shift toward a type 2 immune environment, driven by ILC2s and their associated cytokines, could sustain tumor progression in PCa.

## Discussion

Our understanding on ILCs has evolved significantly in recent years, uncovering previously unrecognized functions. While traditionally associated with defense against helminth infections, ILC2s are now known to play key roles in regulating immune balance, supporting tissue repair, and ensuring the proper function of adipose tissue [65, 66]. Despite these protective functions, ILC2s also play a critical role in promoting tumor growth, with few exceptions [19, 67], in contrast to the predominantly antitumoral activity mediated by ILC1s [18, 20]. Several studies have reported dysfunctional ILC1s in various tumor types [22, 68–70]. Conversely, ILC2s have been described as a protumorigenic subset in CRC, breast, pancreatic, and lung cancer [12, 39, 58, 71]. Importantly, the protumoral role of ILC2s has also been shown in urological cancers, including bladder and testicular cancers, highlighting their potential as therapeutic targets in urological malignancies [12, 72]. In this work, we found that ILC2s and the ILC2-activating cytokines IL-33 and IL-18 are increased both in the tumor microenvironment and in the circulation of patients with PCa, suggesting that, also in prostate cancer, one of the most prevalent forms of cancer in men worldwide, they exert a protumoral function. In this context, a previous enrichment of ILC2s has been identified, along with a positive correlation with the presence of myeloid-derived suppressor cells (MDSCs), supporting the concept of a protumorigenic role for ILC2s [30, 31]. In line with these results, we confirmed the enrichment of ILC2s in a large cohort of patients with PCa in a Gleason score-dependent manner and demonstrated that ILC2s were hyperactivated, producing high levels of IL-13 and IL-5. Recent studies have indicated a potential correlation between the frequency of certain immune cell populations and PSA levels in patients with PCa. Elevated PSA levels have been associated with alterations in immune cell profiles, including increased regulatory T cells (Tregs) and MDSCs, suggesting a possible immunosuppressive microenvironment that may contribute to disease progression and evasion of immune surveillance in PCa [73]. In our setting, we found that the frequency of ILC2s was increased in patients with PSA values higher than 10 ng/ml. Interestingly, since ILC2s have been shown to promote the activity of both MDSCs and Tregs in leukemia and gastric and bladder cancers [17, 28, 30, 74], they could potentially act as upstream players involved in the correlation between these two populations and PSA levels in patients with PCa. Nevertheless, since the use of PSA alone sometimes leads to unnecessary biopsies [75], further studies are needed to clarify the possible correlation between the frequency of ILC2s and PSA values with the other recently developed screening blood-based tests, such as the PHI, 4K score, and Stockholm 3 [8]. Moreover, further additional studies should explore the relationship between ILC2s and chemotherapy resistance in PCa as previously described in CRC patients [76]. Alarmins, also known as damage-associated molecular patterns (DAMPs), play crucial roles in cancer initiation, progression, and metastasis. Among these, IL-33 is a key player in cancer biology and is involved in various aspects of tumor development by modulating the immune response. The IL-33/ILC2 axis plays a multifaceted role in tumor development and progression in different types of cancer. Upon release by both tumor and stromal cells, IL-33 activates ILC2s through its receptor, ST2, leading to the production of cytokines such as IL-5 and IL-13 [77]. More recently, the IL-18/ILC2 axis has also emerged in cancer research, shaping the immune landscape within the tumor microenvironment in CRC, gastric cancer, and CML [16, 31, 39]. We observed that both IL-33 and IL-18 were highly concentrated in the serum of patients with PCa, suggesting that increased exposure of ILC2s to these cytokines may trigger their proliferation and activity, producing high levels of IL-13. Likewise, the frequency and activity (through the secretion of TNF-α and IFN-γ) of ILC1s were impaired in patients with PCa. Notably, unlike ILC2s, ILC1s were proposed as major players in antitumor immunity, showing reduced frequency or an exhausted function in different types of cancer including melanoma, CRC, breast cancer, and AML [20, 22, 78]. One mechanism implicated in the impairment of ILC1s is attributed to ILC1–ILC2 plasticity mediated by different cytokines, including IL-4 [79, 80]. Our results showed that IL-4 is increased in the sera of patients with PCa, suggesting a potential involvement in the ILC1–ILC2 ,conversion as previously described [40, 79–82]. However, additional and more focused studies on ILC1 exhaustion and plasticity are needed to elucidate the involvement of IL-4 and other cytokines in the context of PCa. Finally, incubation of PBMCs from HDs with the CM of PC3 PCa cells, which secrete the observed ILC2-activating cytokines, recapitulated the observations of ILC2s of patients with PCa. Further reinforcing these observations, we revealed a crucial role of ILC2-derived IL-13 in promoting the migration and invasion of PCa cells. The connection between IL-13 and cancer cell migration and invasion has been increasingly explored, with IL-13 identified as a key player in several cancers, including prostate cancer [39, 53, 83]. Our data support these findings, showing that the presence of IL-13, specifically derived from ILC2s, significantly enhances the migratory and invasive behavior of PCa cells, underscoring the potential of targeting this cytokine pathway for therapeutic intervention in PCa. Moreover, the identification by histology and transcriptomics analysis of ILC2s and of their activating cytokines in PCa tissue samples further supports their potential role in shaping the immune environment in this type of cancer. Nevertheless, flow cytometry analysis would be more informative and better suited for phenotyping and quantifying tissue ILC2s, given the need to use multiple markers to characterize these cells. However, this requires freshly collected PCa tissue samples, which are challenging to obtain owing to the prioritization of specimens for clinical diagnostics, which strongly limits the availability of leftover PCa tissues for research purposes. Likewise, we acknowledge that the identification of ILC2s in the scRNA-seq data was based on PTPRC, CD3, and GATA3 markers without the application of gene signature scoring methods, which could enhance the accuracy of our analysis. Despite these constraints, our findings offer a valuable preliminary approach and may serve as a useful starting point for future studies on ILC2s in PCa tissue. Taken together, our results suggest that, in PCa, ILC2s support a protumoral microenvironment at the expense of the antitumoral ILC1s. However, the potential imbalance between ILC1 and ILC2 needs to be further confirmed through additional studies focusing on ILC plasticity and developmental shifts in the PCa microenvironment. The protumoral microenvironment is sustained by IL-33, IL-18, and PGD2, which are, at least in part, secreted by PCa cancer cells supporting the recruitment, proliferation, and function of ILC2s. ILC2 accumulation and hyperactivation result in the hyperproduction of IL-13, which in turn may support PCa cell survival and aggressiveness, as well as MDSC accumulation and activity, as previously described [30, 83]. Thus, ILC2s may represent an additional promising molecular predictor for PCa diagnosis and prognosis and could be exploited as novel therapeutic targets for the treatment of patients with PCa.

## Supplementary Information

Additional file 1.

Additional file 2.

## Data Availability

The data generated in this study will be available from the corresponding author upon request.

## Abbreviations

AML: Acute myelocytic leukemia
APL: Acute promyelocytic leukemia
AREG: Amphiregulin
BPH: Benign prostatic hyperplasia
CM: Conditioned medium
CML: Chronic myeloid leukemia
CRC: Colorectal cancer
DAMPs: Damage-associated molecular patterns
DFS: Disease-free survival
DMSO: Dimethylsulfoxide
FBS: Fetal bovine serum
HDs: Healthy donors
HG: Patients with high-grade cancer
IFN-γ: Interferon-gamma
IL: Interleukin
ILCs: Innate lymphoid cells
ILCPs: ILC precursors
LG: Patients with low-grade cancer
MDSCs: Myeloid-derived suppressor cells
PB: Peripheral blood
PBMCs: Peripheral blood mononuclear cells
PCa: Prostate cancer
PGD2: Prostaglandin D2
PHI: Prostate Health Index
PSA: Prostate-specific antigen
Tregs: Regulatory T cells
TNF-α: Tumor necrosis factor-alpha
TSLP: Thymic stromal lymphopoietin
