# Supplementary material for: Circulating innate lymphoid cells are dysregulated in patients with prostate cancer
Source: Cell Mol Biol Lett. 2025 Apr 18;30:48. doi: 10.1186/s11658-025-00725-7 (PMC12007220; doi:10.1186/s11658-025-00725-7)
Supplement: Supplementary file 1 — Additional file 1. [file 11658_2025_725_MOESM1_ESM.docx]

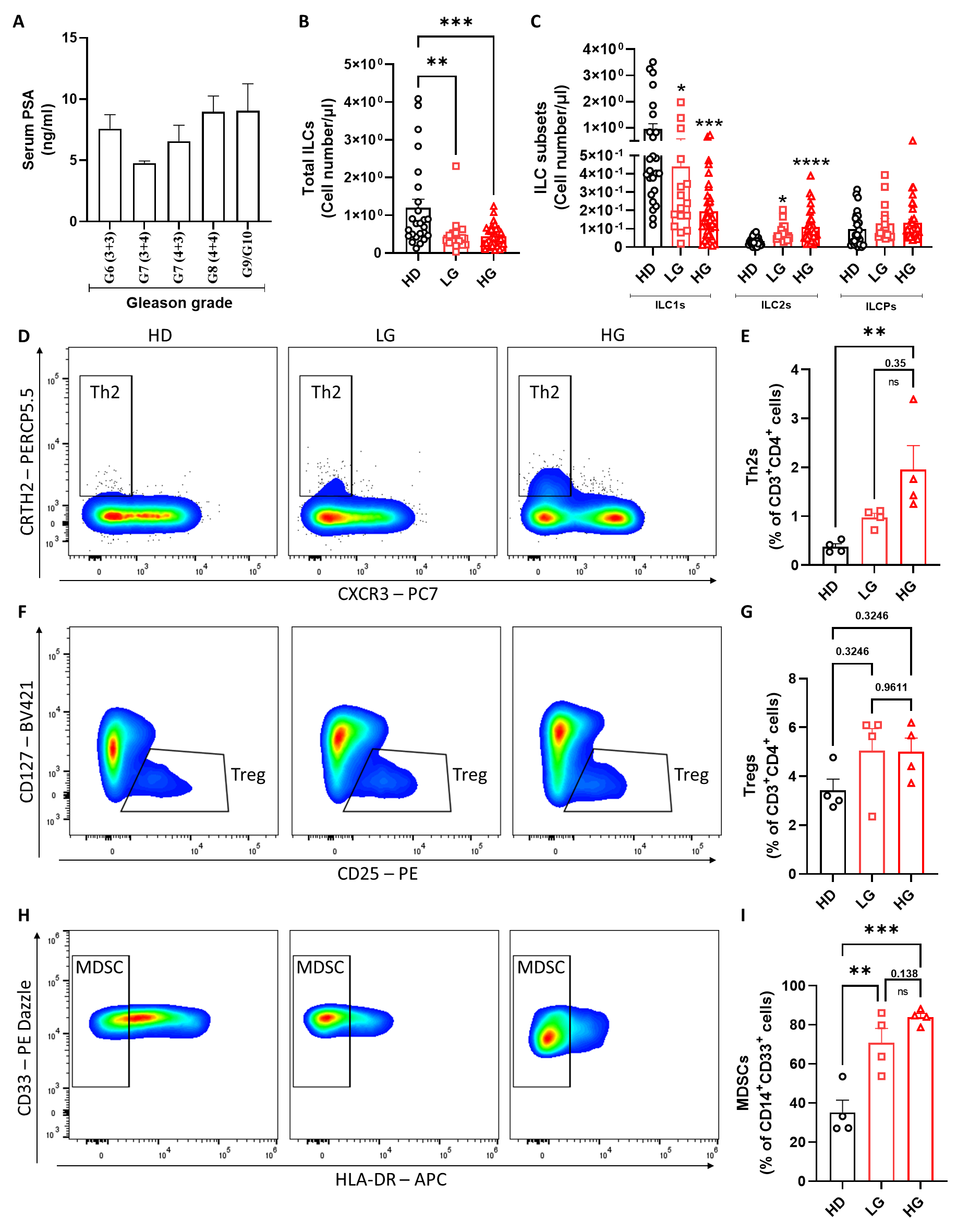


**Supplementary Figure 1.** (**A**) Correlation between PSA levels and Gleason grade in PCa patients (n = 48). (**B, C**) Absolute count measurements of total ILCs (**B**) and ILC subsets (**C**) in HD, LG and HG PCa patients. **(D, F, H)** Representative examples of flow cytometry analysis of Th2s (**D**), Tregs (**F**) and MDSCs (**H**) in HD, LG and HG PCa patients’ PBMC (n= 4). **(E, G, I)** Frequency of Th2s (**E**), Tregs (**G**) and MDSCs (**I**). Data are shown as mean ± SEM (* p < 0.05; ** p < 0.01; ***p < 0.001; ****p < 0.0001) and were analysed by Wilcoxon and/or one-way ANOVA tests.


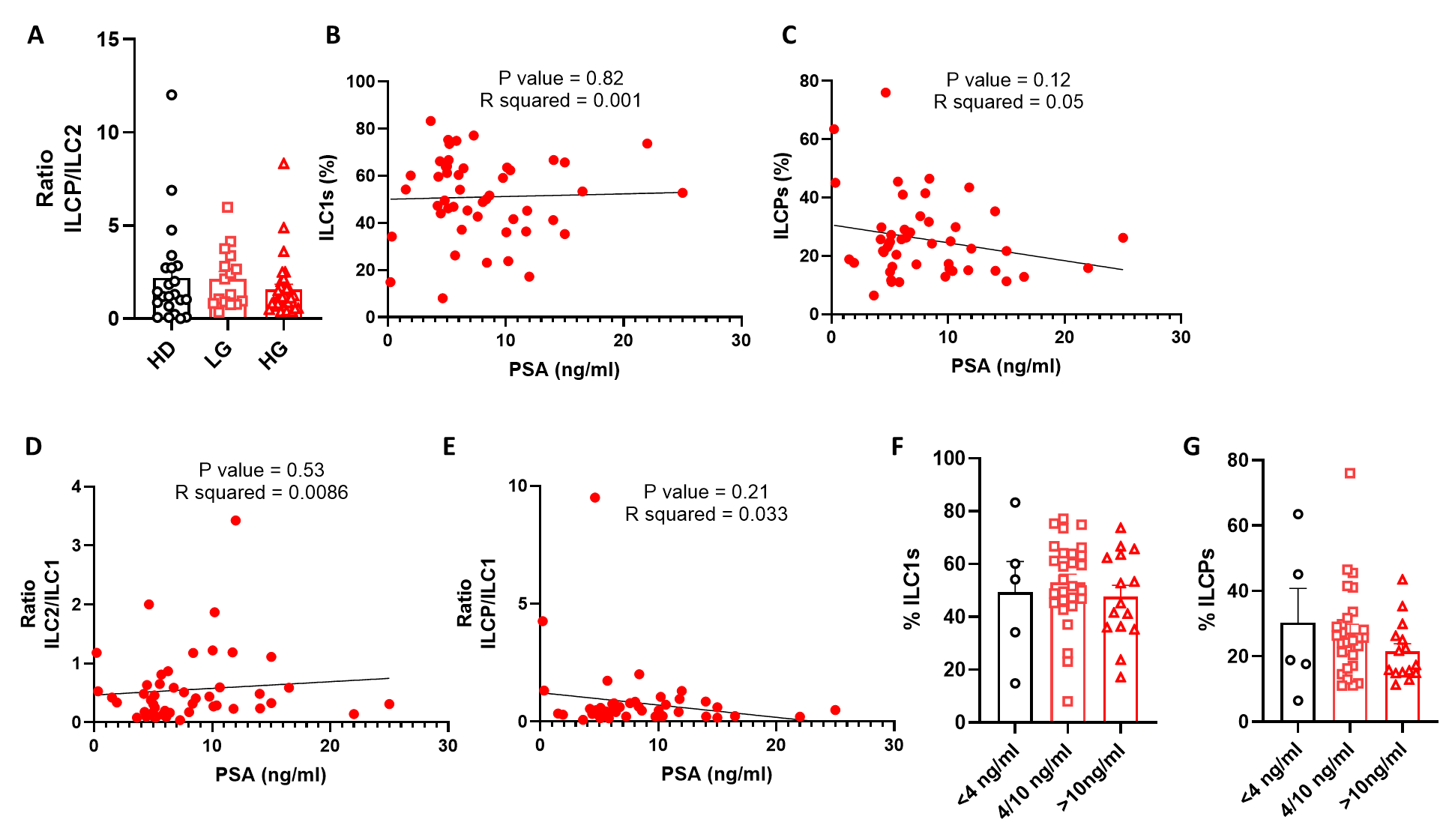


**Supplementary Figure 2. (A)** Ratio of ILCP/ILC2 in HD, LG and HG PCa patients. (**B, C**) Correlations between circulating ILC1 (**B**) and ILCP (**C**) frequency and PSA value (n= 48). **(D, E)** Correlations between ILC2/ILC1 (**D**) and ILCP/ILC1 (**E**) ratios and PSA value (n= 48). (**F, G**) Frequency of ILC1s (**F**) and ILCPs (**G**) based on PSA levels (<4 (n= 5), 4-10 (n = 28) and >10 ng/ml (n= 15), according to the National Comprehensive Cancer Network guidelines.


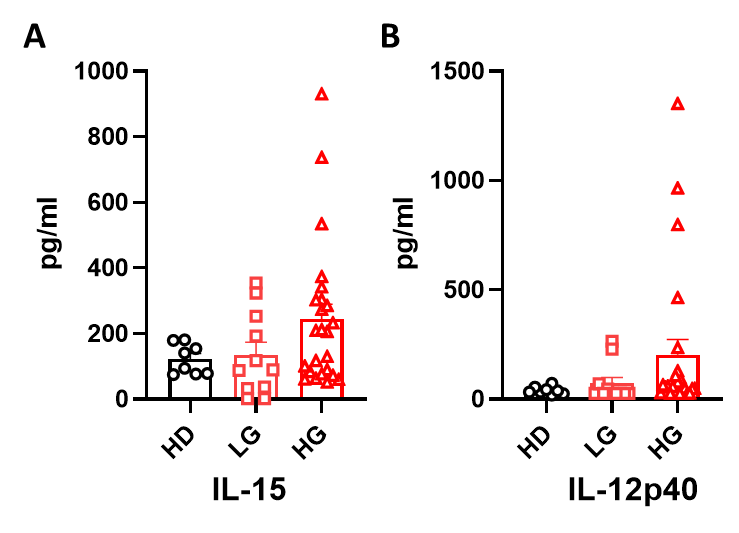


**Supplementary Figure 3.** IL-15 (**A**) and IL-12p40 (**B**) concentrations (pg/ml) in HDs’ (n = 8) LG (n = 11) and HG (n = 24) PCa patients’ sera.


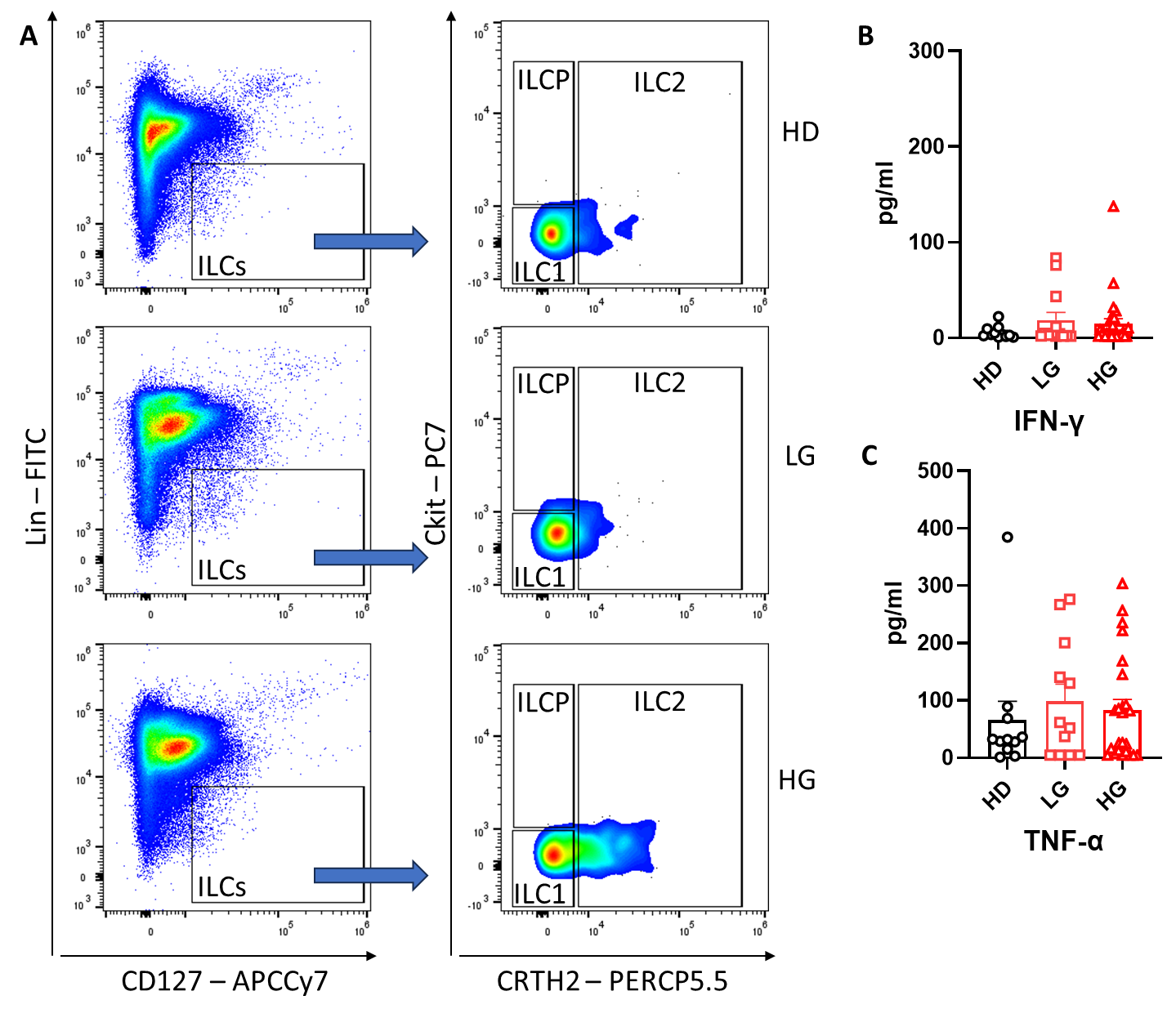


**Supplementary Figure 4.** (**A**) Representative examples of flow cytometry analysis of total ILCs and ILC subsets in HD, LG and HG PCa patients’ PBMC after in vitro stimulation. IFN-γ (**B**) and TNF-α (**C**) concentrations (pg/ml) in HDs’ (n = 11) and LG (n = 12) and HG (n = 24) PCa patients’sera.


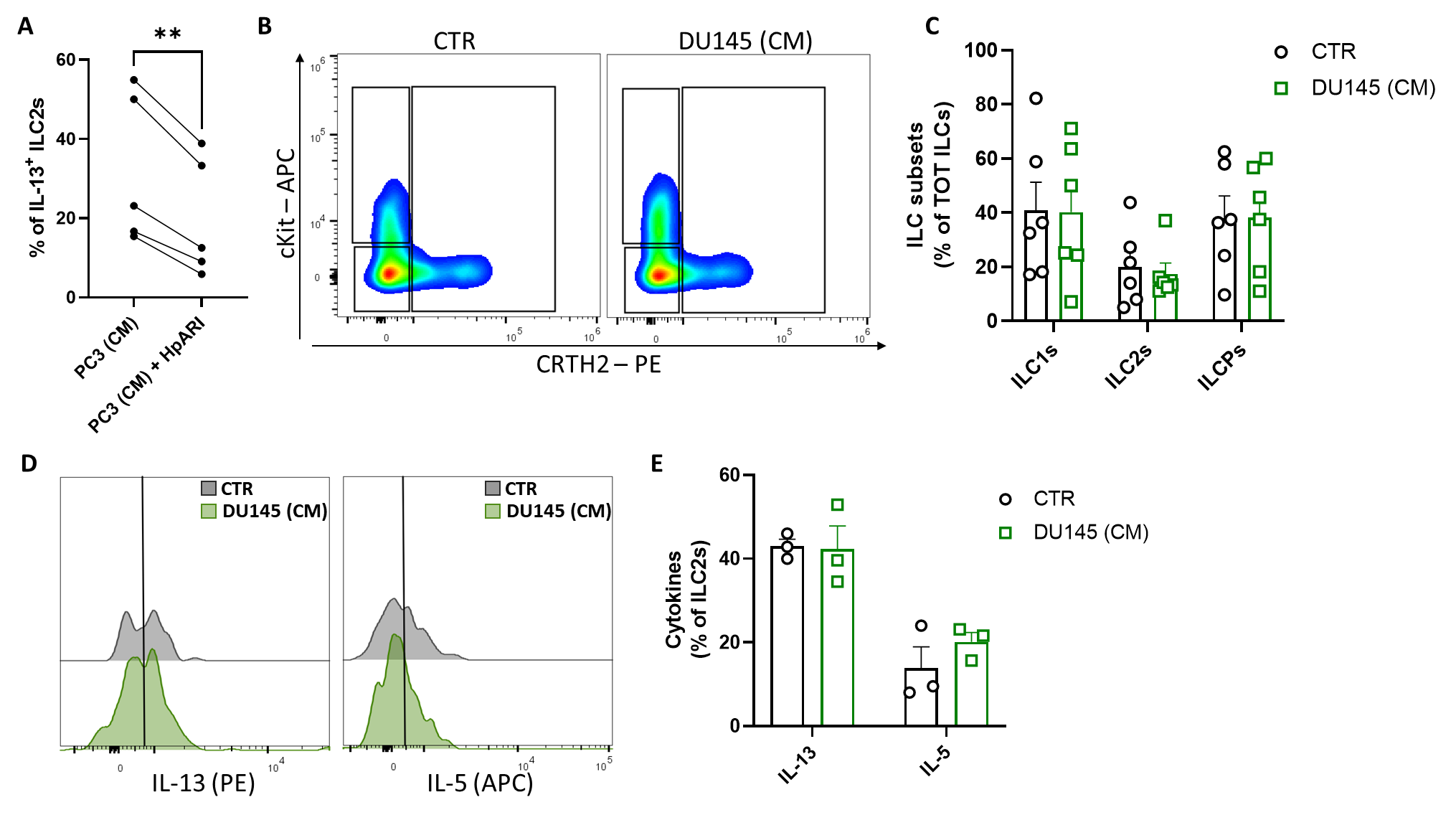


**Supplementary Figure 5. (A)** Frequencies of IL-13 positive ILC2s upon incubation with PC3 conditioned medium (CM), in the presence or absence of HpARI. (**B**) Representative example of flow cytometry analysis of ILC subsets in HD PBMCs cultivated with or without DU145 conditioned media (CM). **(C)** Frequency of ILC subsets in HD PBMCs incubated with or without DU145 CM (n = 6). **(D)** Representative example of flow cytometry analysis of IL-13 and IL-5 positive cell population in HD PBMCs incubated with or without DU145 CM. **(E)** Frequencies of IL-13 and IL-5 positive ILC2s in *ex-vivo* PBMCs upon incubation with or without DU145 CM (n = 3). Data are shown as mean ± SEM (**, P < 0.01) and were analysed by Wilcoxon tests.
