# Supplementary material for: Circulating innate lymphoid cells are dysregulated in patients with prostate cancer
Source: Cell Mol Biol Lett. 2025 Apr 18;30:48. doi: 10.1186/s11658-025-00725-7 (PMC12007220; doi:10.1186/s11658-025-00725-7)
Supplement: Supplementary file 2 — Additional file 2. [file 11658_2025_725_MOESM2_ESM.docx]

**Supplementary Table 1.** Clinical characteristics of healthy donors.
